# Supplementary material for: The Evolution and Characterization of the RNA Interference Pathways in Lophotrochozoa
Source: Genome Biol Evol. 2024 May 7;16(5):evae098. doi: 10.1093/gbe/evae098 (PMC11114477; doi:10.1093/gbe/evae098)
Supplement: evae098_Supplementary_Data [file evae098_supplementary_data.zip › Supplementary_materials.zip!/selected_miRNAs/NoGe_selected_miRNAs/mirdeep_pdf_files/NMRB01003515.1_Notospermus_geniculatus_strain_Ushimado_scaffold3515,_whole_genome_shotgun_sequence_43828.pdf]

Provisional ID : NMRB01003515.1_Notospermus_geniculatus_strain_Ushimado_scaffold3515,_whole_genome_shotgun_sequence_43828
Score total : 156656.2
Score for star read(s) : 3.9
Score for read counts : 156647.5
Score for mfe : 1.9
Score for randfold : 0
Score for cons. seed : 3
Total read count : 307269
Mature read count : 307235
Loop read count : 0
Star read count : 34

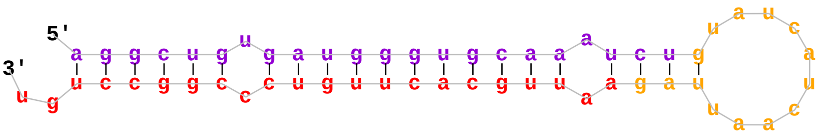

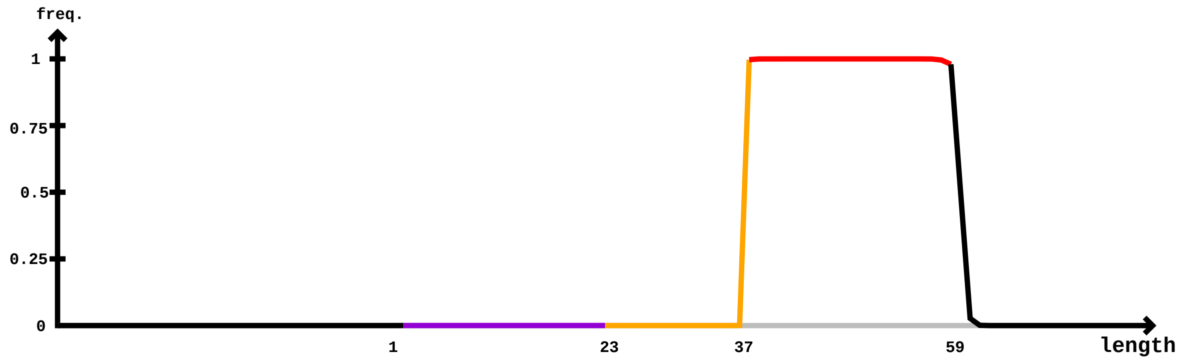

Star Mature

```
5'- uuggaguuggacuuucacuuuugucauguugggcaggcugugaugggugcaaaucuguaucaucaauuagaauugcacuugucccggccuguuugauugacugcaguucauc  -3'  obs
    uuggaguuggacuuucacuuuugucauguugggcaggcugugaugggugcaaaucuguaucaucaauuagaauugcacuugucccggccuguuugauugacugcaguucauc        exp
    .......((((((..((.....((((.(((((((((((((.(((((((((((.((((..........)))).))))))))))).))))))))))))))))))).))))))..   reads   mm   sample
    ..................................aggcugugaugggugcaaaucu........................................................   12      0    Mm1
    .....................................................................Caauugcacuugucccggccugu....................   4       1    Mm1
    ......................................................................aauugcacuugucccggcc.......................   25      0    Mm1
    ......................................................................aauugcacuugucAcggcc.......................   1       1    Mm1
    ......................................................................aauugcacuugucccggGc.......................   1       1    Mm1
    ......................................................................aauugcacuugucccggcUu......................   1       1    Mm1
    ......................................................................aauugcacuAgucccggccu......................   1       1    Mm1
    ......................................................................aauugcacuugucccgAccu......................   2       1    Mm1
    ......................................................................aauugcacuugucccggAcu......................   1       1    Mm1
    ......................................................................aauugcacuugucccggccu......................   140     0    Mm1
    ......................................................................aauugcacuugucccggUcu......................   1       1    Mm1
    ......................................................................aauugcacuuAucccggccu......................   2       1    Mm1
    ......................................................................aauugcacuugucccgAccug.....................   2       1    Mm1
    ......................................................................Uauugcacuugucccggccug.....................   2       1    Mm1
    ......................................................................aauugcacuugucccggUcug.....................   2       1    Mm1
    ......................................................................aauugcacuuAucccggccug.....................   1       1    Mm1
    ......................................................................aauugcacuugucccggccug.....................   527     0    Mm1
    ......................................................................aauugcacuugucccgUccug.....................   1       1    Mm1
    ......................................................................aauugcacuugucccggccuA.....................   6       1    Mm1
    ......................................................................aauugcacuugucccggccuU.....................   2       1    Mm1
    ......................................................................aauugcacuugucccggGcug.....................   5       1    Mm1
    ......................................................................aauugcacuugucAcggccug.....................   1       1    Mm1
    ......................................................................aauugcacuugucccggAcug.....................   2       1    Mm1
    ......................................................................aauugcacuugucccgCccug.....................   1       1    Mm1
    ......................................................................aauugcacuAgucccggccug.....................   1       1    Mm1
    ......................................................................aauugcacuugucccUgccugu....................   4       1    Mm1
    ......................................................................aauugcacuugucccggccugA....................   25      1    Mm1
    ......................................................................aauugcacuugucccggcUugu....................   4       1    Mm1
    ......................................................................aCuugcacuugucccggccugu....................   1       1    Mm1
    ......................................................................aauugNacuugucccggccugu....................   3       1    Mm1
    ......................................................................aauugcacuugucccggcAugu....................   3       1    Mm1
    ......................................................................Cauugcacuugucccggccugu....................   5       1    Mm1
    ......................................................................aauugcUcuugucccggccugu....................   9       1    Mm1
```

```
                                    Star                                   Mature
uuggaguuggacuuucacuuuugucauguugggcaggcugugaugggugcaaaucuguaucaucaauuagaauugcacuugucccggccuguuugauugacugcaguucauc
......................................................................aauugcaGuugucccggccugu....................   1       1   Mm1
......................................................................aauugcacuGgucccggccugu....................   2       1   Mm1
......................................................................aauugcacuugucccggccuCu....................   1       1   Mm1
......................................................................aauugcacuugucccggccugu....................   29917   0   Mm1
......................................................................aauGgcacuugucccggccugu....................   1       1   Mm1
......................................................................aauugcacuuguUccggccugu....................   4       1   Mm1
......................................................................aauugcacuuUucccggccugu....................   24      1   Mm1
......................................................................Uauugcacuugucccggccugu....................   54      1   Mm1
......................................................................aauugcNcuugucccggccugu....................   2       1   Mm1
......................................................................aauugcacuugucccggccuUu....................   6       1   Mm1
......................................................................aauugcacuugAcccggccugu....................   3       1   Mm1
......................................................................aUuugcacuugucccggccugu....................   12      1   Mm1
......................................................................aauugcCcuugucccggccugu....................   1       1   Mm1
......................................................................aauugcacuuCucccggccugu....................   27      1   Mm1
......................................................................aauugcacuuAucccggccugu....................   35      1   Mm1
......................................................................aauugcacuugCcccggccugu....................   8       1   Mm1
......................................................................aauugcacuugucccgAccugu....................   96      1   Mm1
......................................................................aauugcacuugucccggGcugu....................   106     1   Mm1
......................................................................aauCgcacuugucccggccugu....................   5       1   Mm1
......................................................................aauugcacuugucccgCccugu....................   70      1   Mm1
......................................................................aauugcacuugucccggUcugu....................   181     1   Mm1
......................................................................aauugcacuugucAcggccugu....................   5       1   Mm1
......................................................................aaGugcacuugucccggccugu....................   5       1   Mm1
......................................................................aauugcGcuugucccggccugu....................   35      1   Mm1
......................................................................aauugAacuugucccggccugu....................   2       1   Mm1
......................................................................aauugcacuugucccgUccugu....................   49      1   Mm1
......................................................................aauugcacuuguccGggccugu....................   3       1   Mm1
......................................................................aaAugcacuugucccggccugu....................   9       1   Mm1
......................................................................aauugcacuugucccggccugC....................   36      1   Mm1
......................................................................aauugcacAugucccggccugu....................   2       1   Mm1
......................................................................aauAgcacuugucccggccugu....................   2       1   Mm1
......................................................................aauugcacuugucccAgccugu....................   1       1   Mm1
......................................................................aauugcacuugucccggccGgu....................   1       1   Mm1
......................................................................aauugcacuugGcccggccugu....................   1       1   Mm1
......................................................................aauugcacuuguccUggccugu....................   7       1   Mm1
......................................................................aauugcacuCgucccggccugu....................   5675    1   Mm1
......................................................................aauugcacuugucGcggccugu....................   1       1   Mm1
......................................................................aauugcacuugucUcggccugu....................   7       1   Mm1
......................................................................aauugcacuugucccggccuAu....................   6       1   Mm1
......................................................................aauugcacuugucccggccCgu....................   2       1   Mm1
......................................................................aauugcaUuugucccggccugu....................   7       1   Mm1
......................................................................aauuAcacuugucccggccugu....................   1       1   Mm1
......................................................................aauugcacCugucccggccugu....................   3       1   Mm1
......................................................................aauugcacGugucccggccugu....................   1       1   Mm1
......................................................................Gauugcacuugucccggccugu....................   7       1   Mm1
......................................................................Nauugcacuugucccggccugu....................   16      1   Mm1
......................................................................aauugcacuugucccggccAgu....................   1       1   Mm1
......................................................................aauugcacuugucccggcGugu....................   1       1   Mm1
......................................................................aauuUcacuugucccggccugu....................   2       1   Mm1
......................................................................aauugcaAuugucccggccugu....................   2       1   Mm1
......................................................................aauugcacuuguccAggccugu....................   2       1   Mm1
......................................................................aauugcacuuguGccggccugu....................   1       1   Mm1
......................................................................aauugGacuugucccggccugu....................   1       1   Mm1
......................................................................aauugcacuugucccggccugG....................   77      1   Mm1
......................................................................aauugUacuugucccggccugu....................   4       1   Mm1
......................................................................aauugcacuugucccggAcugu....................   243     1   Mm1
......................................................................aauuCcacuugucccggccugu....................   4       1   Mm1
......................................................................aGuugcacuugucccggccugu....................   16      1   Mm1
......................................................................aauugcacuugucccggccuguu...................   1742    0   Mm1
......................................................................aauugcacuugucccggGcuguu...................   4       1   Mm1
......................................................................aauugcacuugucccgCccuguu...................   7       1   Mm1
......................................................................aauugcacuugucccggccuguG...................   6       1   Mm1
......................................................................aauugcacuCgucccggccuguu...................   400     1   Mm1
......................................................................aauugcacuugucUcggccuguu...................   1       1   Mm1
......................................................................aauugcacuuguUccggccuguu...................   1       1   Mm1
......................................................................aauugcacuuAucccggccuguu...................   2       1   Mm1
......................................................................aauugcacuugucccgAccuguu...................   3       1   Mm1
......................................................................aauugcacuugucccggUcuguu...................   5       1   Mm1
......................................................................aauugcacuugucccgUccuguu...................   4       1   Mm1
......................................................................aauugcacuugucccggcCguu...................   1       1   Mm1
```

```
                                  Star                                Mature
uuggaguuggacuuucacuuuugucauguugggcaggcugugaugggugcaaaucuguaucaucaauuagaauugcacuugucccggccuguuugauugacugcaguucauc
......................................................................aauugcacuugucccggAcuguu...................   19    1   Mm1
......................................................................aaAugcacuugucccggccuguu...................   1     1   Mm1
......................................................................aauAgcacuugucccggccuguu...................   2     1   Mm1
......................................................................aauugcGcuugucccggccuguu...................   1     1   Mm1
......................................................................aauugcacuugucccggccugAu...................   1     1   Mm1
......................................................................aauugNacuugucccggccuguu...................   1     1   Mm1
......................................................................aGuugcacuugucccggccuguu...................   3     1   Mm1
......................................................................aauugcacuuguccAggccuguu...................   1     1   Mm1
......................................................................aauugcacuugucccggccuguA...................   1363  1   Mm1
......................................................................aCuugcacuugucccggccuguu...................   1     1   Mm1
......................................................................aauugcacuugucAcggccuguu...................   1     1   Mm1
......................................................................aauugcacuuCucccggccuguu...................   4     1   Mm1
......................................................................aauugcaUuugucccggccuguu...................   1     1   Mm1
......................................................................Uauugcacuugucccggccuguu...................   3     1   Mm1
......................................................................Cauugcacuugucccggccuguu...................   1     1   Mm1
......................................................................aauugcacuugucccggcAuguuu..................   1     1   Mm1
......................................................................aauugcacuugucccggccuguAu..................   310   1   Mm1
......................................................................aauugcacuugucccggccuguCu..................   1     1   Mm1
......................................................................aauugcacuugucccggccuguuA..................   1     1   Mm1
......................................................................aauugcacuCgucccggccuguuu..................   75    1   Mm1
......................................................................aauugcacuugucccggUcuguuu..................   1     1   Mm1
......................................................................aauugcacuuguccUggccuguuu..................   7     1   Mm1
......................................................................aauugcacuuCucccggccuguuu..................   1     1   Mm1
......................................................................aauugcacuugucccggccuguuu..................   254   0   Mm1
......................................................................aauugcacuugucccggGcuguuu..................   1     1   Mm1
......................................................................aauugcUcuugucccggccuguuu..................   3     1   Mm1
......................................................................aauugcacuugucccggAcuguuu..................   1     1   Mm1
......................................................................aauugcacuugucccggccuguuuU.................   14    1   Mm1
......................................................................aauugcacuugucccggccuguuuUa................   1     1   Mm1
.......................................................................auugcacuugucccggcGugu....................   1     1   Mm1
.......................................................................auugcacuuguUccggccugu....................   1     1   Mm1
.......................................................................auugcacuugucccggccugu....................   48    0   Mm1
.......................................................................auugcacuugCcccggccugu....................   2     1   Mm1
.......................................................................auugcacuuUucccggccugu....................   1     1   Mm1
.......................................................................auugcacuugucccggccugG....................   1     1   Mm1
.......................................................................auugcacuCgucccggccugu....................   26    1   Mm1
.......................................................................auugcacuugucccggccuguA...................   13    1   Mm1
.......................................................................auugcacuugucccggccuguu...................   3     0   Mm1
.......................................................................auugcacuCgucccggccuguu...................   1     1   Mm1
.......................................................................auugcacuugucccggccuguAu..................   14    1   Mm1
.......................................................................auugcacuugucccggccuguuu..................   3     0   Mm1
........................................................................uugcacuugucccggcUugu....................   1     1   Mm1
........................................................................uugcacuugucccggccugu....................   6     0   Mm1
..........................................................................gcacuugucccggccugu....................   1     0   Mm1

..................................aggcugugaugggugcaaaucu........................................................   3     0   Mm2
..................................aggcugugauAggugcaaaucu........................................................   4     1   Mm2
.....................................................................Caauugcacuugucccggccug.....................   1     1   Mm2
.....................................................................Caauugcacuugucccggccugu....................   39    1   Mm2
.....................................................................Uaauugcacuugucccggccugu....................   1     1   Mm2
.....................................................................Caauugcacuugucccggccuguu...................   2     1   Mm2
......................................................................aauugcacuugucccggc........................   2     0   Mm2
......................................................................aGuugcacuugucccggc........................   1     1   Mm2
......................................................................aauugcacuugAcccggcc.......................   1     1   Mm2
......................................................................aauugcacuugucccggcc.......................   38    0   Mm2
......................................................................Uauugcacuugucccggccu......................   1     1   Mm2
......................................................................aauugcacuAgucccggccu......................   1     1   Mm2
......................................................................aauugcacuuAucccggccu......................   4     1   Mm2
......................................................................aauugcacuugucccggccG......................   9     1   Mm2
......................................................................aauugcacuugucccggAcu......................   2     1   Mm2
......................................................................aauugcacuugucccggUcu......................   1     1   Mm2
......................................................................aauugcacuuCucccggccu......................   7     1   Mm2
......................................................................aauugcacuugucccggccC......................   1     1   Mm2
......................................................................aauugcaUuugucccggccu......................   1     1   Mm2
......................................................................aauugcacuugucccggccu......................   313   0   Mm2
......................................................................aauugcacuugucccggGcug.....................   8     1   Mm2
......................................................................aauugcacuugucccggAcug.....................   25    1   Mm2
......................................................................aauugcacuuAucccggccug.....................   3     1   Mm2
......................................................................aauugcacCugucccggccug.....................   1     1   Mm2
......................................................................Nauugcacuugucccggccug.....................   1     1   Mm2
```

Star Mature

```
uuggaguuggacuuucacuuuugucauguugggcaggcugugaugggugcaaaucuguaucaucaauuagaauugcacuugucccggccuguuugauugacugcaguucauc
......................................................................aauugcacuugucAcggccug.....................  1     1  Mm2
......................................................................aauugcacuugucccgUccug.....................  4     1  Mm2
......................................................................aauugcacuugCcccggccug.....................  1     1  Mm2
......................................................................aauugcacuugucccggccuU.....................  7     1  Mm2
......................................................................aauugcacuugucccgAccug.....................  9     1  Mm2
......................................................................aauugNacuugucccggccug.....................  1     1  Mm2
......................................................................aauugcGcuugucccggccug.....................  2     1  Mm2
......................................................................aauugcacuuguccUggccug.....................  1     1  Mm2
......................................................................aauugcacuugucccgCccug.....................  6     1  Mm2
......................................................................aauugcacuugucccggccCg.....................  1     1  Mm2
......................................................................Uauugcacuugucccggccug.....................  3     1  Mm2
......................................................................aauugcacuugucUcggccug.....................  3     1  Mm2
......................................................................aauugcacuuCucccggccug.....................  1     1  Mm2
......................................................................aauugcacuugucccggccuC.....................  1     1  Mm2
......................................................................aauugcacuugucccggUcug.....................  11    1  Mm2
......................................................................aauugcacuuUucccggccug.....................  1     1  Mm2
......................................................................aauugcacuugucccggccuA.....................  2     1  Mm2
......................................................................aauugcacuugucccggccug.....................  1882  0  Mm2
......................................................................Cauugcacuugucccggccug.....................  1     1  Mm2
......................................................................aauugcacuugucccggccGg.....................  54    1  Mm2
......................................................................aauCgcacuugucccggccugu....................  15    1  Mm2
......................................................................aauugUacuugucccggccugu....................  36    1  Mm2
......................................................................aauugcacuugucccggcUugu....................  25    1  Mm2
......................................................................aauugcacuuAucccggccugu....................  235   1  Mm2
......................................................................aauugcacuugucccggcAugu....................  5     1  Mm2
......................................................................aauugcacuugCcccggccugu....................  11    1  Mm2
......................................................................aauugcacuugucccggGcugu....................  411   1  Mm2
......................................................................aauugcCcuugucccggccugu....................  3     1  Mm2
......................................................................aauGgcacuugucccggccugu....................  5     1  Mm2
......................................................................aauugcacuugucccCgccugu....................  2     1  Mm2
......................................................................aauugcacuugucUcggccugu....................  60    1  Mm2
......................................................................aauugcacuugGcccggccugu....................  2     1  Mm2
......................................................................aCuugcacuugucccggccugu....................  11    1  Mm2
......................................................................aauugcacuugucccggccuCu....................  50    1  Mm2
......................................................................aauuUcacuugucccggccugu....................  17    1  Mm2
......................................................................aauugcacuugucccAgccugu....................  25    1  Mm2
......................................................................aauugcacuGgucccggccugu....................  7     1  Mm2
......................................................................aauugcaGuugucccggccugu....................  4     1  Mm2
......................................................................aauugAacuugucccggccugu....................  8     1  Mm2
......................................................................aauugcNcuugucccggccugu....................  7     1  Mm2
......................................................................aauuAcacuugucccggccugu....................  21    1  Mm2
......................................................................aauugcacAugucccggccugu....................  8     1  Mm2
......................................................................aauugcacuugucccgAccugu....................  293   1  Mm2
......................................................................aauugcacuuUucccggccugu....................  113   1  Mm2
......................................................................aauugcacuuguccUggccugu....................  40    1  Mm2
......................................................................aauugNacuugucccggccugu....................  13    1  Mm2
......................................................................aauugcacuuguAccggccugu....................  11    1  Mm2
......................................................................aauugcaUuugucccggccugu....................  18    1  Mm2
......................................................................aauugcUcuugucccggccugu....................  19    1  Mm2
......................................................................aauugcacuugucccggccuAu....................  12    1  Mm2
......................................................................aauugcacuugucccggccugG....................  1539  1  Mm2
......................................................................aauugcacuugucccggccugC....................  204   1  Mm2
......................................................................aauugcacuugucccggAcugu....................  1015  1  Mm2
......................................................................aauugcacuugucccggccugA....................  46    1  Mm2
......................................................................aauugcacuugucccggccGgu....................  5     1  Mm2
......................................................................aauugcacuugucccggccAgu....................  5     1  Mm2
......................................................................aauugcacuuguccAggccugu....................  8     1  Mm2
......................................................................aaGugcacuugucccggccugu....................  27    1  Mm2
......................................................................aauugcacuugucccgCccugu....................  295   1  Mm2
......................................................................aauugcacCugucccggccugu....................  13    1  Mm2
......................................................................aauAgcacuugucccggccugu....................  10    1  Mm2
......................................................................aauugcacuuCucccggccugu....................  108   1  Mm2
......................................................................aauugcacuugucAcggccugu....................  31    1  Mm2
......................................................................aauugcacuugucccggccCgu....................  16    1  Mm2
......................................................................aGuugcacuugucccggccugu....................  21    1  Mm2
......................................................................aauugcacuugucccgUccugu....................  226   1  Mm2
......................................................................aauugcacuAgucccggccugu....................  7     1  Mm2
......................................................................aauugcacuugucccggccuUu....................  14    1  Mm2
......................................................................aauugcacuugucccUgccugu....................  14    1  Mm2
......................................................................aaAugcacuugucccggccugu....................  17    1  Mm2
```

```
                                  Star                              Mature
uuggaguuggacuuucacuuuugucauguugggcaggcugugaugggugcaaaucuguaucaucaauuagaauugcacuugucccggccuguuugauugacugcaguucauc
......................................................................Nauugcacuugucccggccugu....................	59	1	Mm2
......................................................................aauugcacuugucccggUcugu....................	589	1	Mm2
......................................................................aaCugcacuugucccggccugu....................	15	1	Mm2
......................................................................aauugcacuuguUccggccugu....................	48	1	Mm2
......................................................................aauugcacuugucccggcGugu....................	3	1	Mm2
......................................................................Gauugcacuugucccggccugu....................	19	1	Mm2
......................................................................aauugcGcuugucccggccugu....................	39	1	Mm2
......................................................................aauugcacuugucccggccugu....................	113962	0	Mm2
......................................................................aauugcacuugAcccggccugu....................	14	1	Mm2
......................................................................aauugcacuuguccGggccugu....................	5	1	Mm2
......................................................................aNuugcacuugucccggccugu....................	1	1	Mm2
......................................................................aauugcacGugucccggccugu....................	1	1	Mm2
......................................................................aUuugcacuugucccggccugu....................	27	1	Mm2
......................................................................Cauugcacuugucccggccugu....................	5	1	Mm2
......................................................................aauuCcacuugucccggccugu....................	11	1	Mm2
......................................................................aauugcacuuguGccggccugu....................	7	1	Mm2
......................................................................aauugcacuugucGcggccugu....................	13	1	Mm2
......................................................................Uauugcacuugucccggccugu....................	97	1	Mm2
......................................................................aauugcaAuugucccggccugu....................	9	1	Mm2
......................................................................aauugcacuCgucccggccugu....................	6825	1	Mm2
......................................................................aauugGacuugucccggccugu....................	2	1	Mm2
......................................................................aauugcacuugucccggAcuguu...................	81	1	Mm2
......................................................................aauugcacuuguUccggccuguu...................	4	1	Mm2
......................................................................aauugcacuuUucccggccuguu...................	8	1	Mm2
......................................................................aauugcGcuugucccggccuguu...................	3	1	Mm2
......................................................................aauugcacuugucccggUcuguu...................	45	1	Mm2
......................................................................aauugcacuugucccggccuguA...................	3949	1	Mm2
......................................................................aauugcacuugucccgCccuguu...................	27	1	Mm2
......................................................................aauugcacuugucccggGcuguu...................	29	1	Mm2
......................................................................aUuugcacuugucccggccuguu...................	2	1	Mm2
......................................................................aauugcacuCgucccggccuguu...................	372	1	Mm2
......................................................................aauugcacuugCcccggccuguu...................	1	1	Mm2
......................................................................aauugAacuugucccggccuguu...................	3	1	Mm2
......................................................................aauugNacuugucccggccuguu...................	3	1	Mm2
......................................................................aauugcacuugucccggccuguG...................	35	1	Mm2
......................................................................aauugcacuugucccggccuCuu...................	2	1	Mm2
......................................................................Gauugcacuugucccggccuguu...................	1	1	Mm2
......................................................................aauugcacuugAcccggccuguu...................	1	1	Mm2
......................................................................aaAugcacuugucccggccuguu...................	1	1	Mm2
......................................................................aauugcUcuugucccggccuguu...................	1	1	Mm2
......................................................................aauugcacuugucccgAccuguu...................	32	1	Mm2
......................................................................aauugcacuugucccggccuguC...................	9	1	Mm2
......................................................................aauugcacuugucccggccuUuu...................	2	1	Mm2
......................................................................aauugcacuuCucccggccuguu...................	5	1	Mm2
......................................................................aauugcacuuguccUggccuguu...................	8	1	Mm2
......................................................................Uauugcacuugucccggccuguu...................	5	1	Mm2
......................................................................Nauugcacuugucccggccuguu...................	2	1	Mm2
......................................................................aauugcacuugucccCgccuguu...................	1	1	Mm2
......................................................................aauCgcacuugucccggccuguu...................	4	1	Mm2
......................................................................aauugcacuAgucccggccuguu...................	1	1	Mm2
......................................................................aauugcaUuugucccggccuguu...................	2	1	Mm2
......................................................................aauugcacuugucccggccuguu...................	9002	0	Mm2
......................................................................aauugcacuugucUcggccuguu...................	6	1	Mm2
......................................................................aauAgcacuugucccggccuguu...................	3	1	Mm2
......................................................................aauugcacuugucccggccCguu...................	1	1	Mm2
......................................................................aauugcacuugucccggccugAu...................	1	1	Mm2
......................................................................aauugcacuugucccggcUuguu...................	2	1	Mm2
......................................................................aauugcacuuAucccggccuguu...................	22	1	Mm2
......................................................................aauugcacuugucccggccugCu...................	3	1	Mm2
......................................................................aauuUcacuugucccggccuguu...................	1	1	Mm2
......................................................................aauugcacuugucccgUccuguu...................	18	1	Mm2
......................................................................aauugUacuugucccggccuguu...................	2	1	Mm2
......................................................................aauugcacuugucccAgccuguu...................	1	1	Mm2
......................................................................aauugcacuuguccAggccuguu...................	3	1	Mm2
......................................................................aaGugcacuugucccggccuguu...................	2	1	Mm2
......................................................................aauugcacuugucccggccugGu...................	3	1	Mm2
......................................................................aauugcacuugucAcggccuguu...................	3	1	Mm2
......................................................................aauugcacuugucccggcAuguu...................	1	1	Mm2
......................................................................aauugcacuuUucccggccuguuu..................	3	1	Mm2
......................................................................aauugcacuugucccggccCguuu..................	1	1	Mm2
```

```
                                  Star                                 Mature
uuggaguuggacuuucacuuuugucauguugggcaggcugugaugggugcaaaucuguaucaucaauuagaauugcacuugucccggccuguuugauugacugcaguucauc
......................................................................aauugcacuGgucccggccuguuu..................   1      1    Mm2
......................................................................aauugcacuugucccggccuguCu..................   7      1    Mm2
......................................................................aauugcaUuugucccggccuguuu..................   1      1    Mm2
......................................................................aauugcacuuguGccggccuguuu..................   1      1    Mm2
......................................................................aauugcacuCgucccggccuguuu..................   86     1    Mm2
......................................................................aauugcacuugucccggGcuguuu..................   5      1    Mm2
......................................................................aauugcacuugucccgAccuguuu..................   7      1    Mm2
......................................................................Nauugcacuugucccggccuguuu..................   2      1    Mm2
......................................................................aauugcacuugucccggccuguuG..................   4      1    Mm2
......................................................................aauugcacuugucccggccuguuA..................   48     1    Mm2
......................................................................aauugcacuugucccggccuguGu..................   2      1    Mm2
......................................................................aauugcacuugucccggccuguuu..................   1557   0    Mm2
......................................................................aauugcacuugucccggccuguAu..................   1211   1    Mm2
......................................................................aauugcacuugucccggUcuguuu..................   9      1    Mm2
......................................................................aauugcacuugucccggccuguuC..................   5      1    Mm2
......................................................................Uauugcacuugucccggccuguuu..................   2      1    Mm2
......................................................................aauugcacuugucUcggccuguuu..................   2      1    Mm2
......................................................................aauugcacuugucccggAcuguuu..................   9      1    Mm2
......................................................................aauugcaAuugucccggccuguuu..................   2      1    Mm2
......................................................................aauuAcacuugucccggccuguuu..................   1      1    Mm2
......................................................................aauugcacuugucccggccuUuuu..................   2      1    Mm2
......................................................................aauugcacuuCucccggccuguuu..................   1      1    Mm2
......................................................................aauugcacuugucccgUccuguuu..................   4      1    Mm2
......................................................................aauugcacuuAucccggccuguuu..................   4      1    Mm2
......................................................................aauugcacuugucccggccuCuuu..................   1      1    Mm2
......................................................................aauCgcacuugucccggccuguuu..................   1      1    Mm2
......................................................................aaAugcacuugucccggccuguuu..................   1      1    Mm2
......................................................................aauugcacuugucccggccuguuuU.................   140    1    Mm2
......................................................................aauugcacuugucccggccuguuuA.................   1      1    Mm2
......................................................................aauugcacuugucccggccuguuug.................   2      0    Mm2
......................................................................aauugcacuugucccggccuguAug.................   1      1    Mm2
......................................................................aauugcacuugucccggccuguuuC.................   1      1    Mm2
.......................................................................auugcacuuguUccggccug.....................   1      1    Mm2
.......................................................................auugcacuugucccggccug.....................   8      0    Mm2
.......................................................................auugcacuGgucccggccugu....................   1      1    Mm2
.......................................................................auugcacuugucccggccugG....................   3      1    Mm2
.......................................................................auugcacuuUucccggccugu....................   5      1    Mm2
.......................................................................auugcacuCgucccggccugu....................   22     1    Mm2
.......................................................................auugcacuugGcccggccugu....................   2      1    Mm2
.......................................................................auugcacuugucccggccuUu....................   2      1    Mm2
.......................................................................auugcacuugucccggAcugu....................   1      1    Mm2
.......................................................................auugcacuugucccggccCgu....................   2      1    Mm2
.......................................................................auugcacuugucccggccugu....................   201    0    Mm2
.......................................................................auugGacuugucccggccugu....................   1      1    Mm2
.......................................................................auugcacuuguUccggccugu....................   2      1    Mm2
.......................................................................auuUcacuugucccggccugu....................   1      1    Mm2
.......................................................................auuAcacuugucccggccugu....................   2      1    Mm2
.......................................................................auuUcacuugucccggccuguu...................   1      1    Mm2
.......................................................................auugcacuugucccggccuguA...................   42     1    Mm2
.......................................................................auugcacuCgucccggccuguu...................   2      1    Mm2
.......................................................................auugcacuuUucccggccuguu...................   1      1    Mm2
.......................................................................auugcacuugucccggccuguu...................   20     0    Mm2
.......................................................................auugcacuugucccggccuguuu..................   6      0    Mm2
.......................................................................auugcacuugucccggccuguuA..................   1      1    Mm2
.......................................................................auugcacuCgucccggccuguuu..................   2      1    Mm2
.......................................................................auugcacuugucccggccuguAu..................   10     1    Mm2
........................................................................uugcacuugucccggccugu....................   4      0    Mm2
.........................................................................ugcacuugucccggccuCu....................   1      1    Mm2
.........................................................................ugcacuugucccggccugu....................   3      0    Mm2
..........................................................................gcacuugucccggccugu....................   4      0    Mm2

......................................................................aauugcacuuCucccggccu......................   1      1    Fm2
......................................................................aauugcacuuAucccggccu......................   1      1    Fm2
......................................................................aauugcacuugucccggccG......................   4      1    Fm2
......................................................................aauugcacuugucccggccu......................   56     0    Fm2
......................................................................aauugcacuugucccggccA......................   1      1    Fm2
......................................................................aauugcacuugucccggAcu......................   1      1    Fm2
......................................................................aauugcacuugucccggAcug.....................   1      1    Fm2
......................................................................aauugcacuugucccggccug.....................   123    0    Fm2
......................................................................aauugcacuugucccggccuA.....................   1      1    Fm2
```

Star **Mature**

```
uuggaguuggacuuucacuuuugucauguugggcaggcugugaugggugcaaaucuguaucaucaauuagaauugcacuugucccggccuguuugauugacugcaguucauc
......................................................................aauugcacuugucccgUccug.....................   1      1   Fm2
......................................................................aauugcacuugucccggUcug.....................   1      1   Fm2
......................................................................aauugcacuuCucccggccug.....................   1      1   Fm2
......................................................................aauugcacuugucccggccGg.....................   8      1   Fm2
......................................................................Uauugcacuugucccggccugu....................   11     1   Fm2
......................................................................aGuugcacuugucccggccugu....................   1      1   Fm2
......................................................................aauugcacuuCucccggccugu....................   2      1   Fm2
......................................................................aauugcacuuAucccggccugu....................   5      1   Fm2
......................................................................aauugcacuugAcccggccugu....................   1      1   Fm2
......................................................................aauugcacuugucccggAcugu....................   18     1   Fm2
......................................................................aauugcacuugucccggccugC....................   6      1   Fm2
......................................................................aauugcGcuugucccggccugu....................   5      1   Fm2
......................................................................Nauugcacuugucccggccugu....................   1      1   Fm2
......................................................................aauugcacuugucccggGcugu....................   3      1   Fm2
......................................................................aauugcacuugucccggccuCu....................   3      1   Fm2
......................................................................aauugcacuugucccgCccugu....................   1      1   Fm2
......................................................................aauugGacuugucccggccugu....................   1      1   Fm2
......................................................................aauugcacuugucccggccugA....................   5      1   Fm2
......................................................................Cauugcacuugucccggccugu....................   1      1   Fm2
......................................................................aauugcacuugucccggUcugu....................   7      1   Fm2
......................................................................aauugcacuugucccgUccugu....................   1      1   Fm2
......................................................................aUuugcacuugucccggccugu....................   1      1   Fm2
......................................................................aauugcacuugucccgAccugu....................   3      1   Fm2
......................................................................aauugcacuCgucccggccugu....................   367    1   Fm2
......................................................................aauugcacuugucccggccuUu....................   1      1   Fm2
......................................................................aauugcacuugucccggccugG....................   34     1   Fm2
......................................................................aauugcacuugucccggccugu....................   1878   0   Fm2
......................................................................aauugcacuugucccggGcuguu...................   2      1   Fm2
......................................................................aauugcacuuUucccggccuguu...................   1      1   Fm2
......................................................................aauugcacuugucccggccuguG...................   1      1   Fm2
......................................................................aauugcaUuugucccggccuguu...................   1      1   Fm2
......................................................................aauugcacuugucccggAcuguu...................   3      1   Fm2
......................................................................aauugcacuugucccggccugCu...................   3      1   Fm2
......................................................................aauugcacuCgucccggccuguu...................   209    1   Fm2
......................................................................aauugcacuugucccggccuUuu...................   1      1   Fm2
......................................................................aauugcacuugucccgUccuguu...................   2      1   Fm2
......................................................................Cauugcacuugucccggccuguu...................   2      1   Fm2
......................................................................aauugcacuugucccggccuguu...................   369    0   Fm2
......................................................................aauugcacuugucccgCccuguu...................   1      1   Fm2
......................................................................aauugcacuugucccggUcuguu...................   1      1   Fm2
......................................................................aauugcacuugucccggccuguA...................   362    1   Fm2
......................................................................aauugcacuugucccUgccuguu...................   1      1   Fm2
......................................................................aauugcacuugucccggccuguuu..................   247    0   Fm2
......................................................................aauugcacuugucccggccuguuG..................   13     1   Fm2
......................................................................aauugcacuugucGcggccuguuu..................   1      1   Fm2
......................................................................aauugcacuugucccggccuguuA..................   46     1   Fm2
......................................................................Nauugcacuugucccggccuguuu..................   1      1   Fm2
......................................................................aauugcacuugucccggccuguAu..................   47     1   Fm2
......................................................................aUuugcacuugucccggccuguuu..................   1      1   Fm2
......................................................................aauugcacuugucccggAcuguuu..................   2      1   Fm2
......................................................................aauugcacuCgucccggccuguuu..................   105    1   Fm2
......................................................................aauugcacuugucccggccuguuug.................   1      0   Fm2
......................................................................aauugcacuugucccggccuguuuU.................   63     1   Fm2
.......................................................................auugcacuugucccggccugu....................   4      0   Fm2
.......................................................................auugcacuugucccggccuguu...................   1      0   Fm2
.......................................................................auugcacuugucccggccuguuu..................   2      0   Fm2
.......................................................................auugcacuugucccggccuUuuu..................   1      1   Fm2

..................................aggcugugaugggugcaaaucu........................................................   2      0   Fm1
..................................aggcugugaugggugcaCaucu........................................................   1      1   Fm1
.....................................................ucuguaucaucaauuagaauugcacuugucccggccugu....................   1      0   Fm1
.....................................................................Caauugcacuugucccggccug.....................   3      1   Fm1
.....................................................................Caauugcacuugucccggccugu....................   15     1   Fm1
.....................................................................Caauugcacuugucccggccuguu...................   2      1   Fm1
......................................................................aauugcacuugucccggcU.......................   5      1   Fm1
......................................................................aauugcacuugucccggccG......................   8      1   Fm1
......................................................................aauugcacuugucccggAcu......................   1      1   Fm1
......................................................................aauugcacuuAucccggccu......................   6      1   Fm1
......................................................................aauugcacuugucccggccu......................   107    0   Fm1
......................................................................aauugcacuAgucccggccug.....................   1      1   Fm1
```

Star / Mature			
uuggaguuggacuuucacuuuugucauguuggcaggcugugaugggugcaaaucuguaucaucaauuagaauugcacuugucccggccuguuugauugacugcaguucauc			
....Uauugcacuugucccggccug.....................	1	1	Fm1
....aauugcGcuugucccggccug.....................	1	1	Fm1
....aauugcacuugucccggccGg.....................	13	1	Fm1
....aauugcacuugucccggUcug.....................	2	1	Fm1
....aauugcacuugucccggAcug.....................	1	1	Fm1
....aauugcacuugucccggGcug.....................	1	1	Fm1
....aauugcacuuguAccggccug.....................	1	1	Fm1
....aauugcacuugucccggccuA.....................	3	1	Fm1
....aauugcacuugucccgUccug.....................	2	1	Fm1
....aaGugcacuugucccggccug.....................	1	1	Fm1
....aauugcacuugucccggccuU.....................	5	1	Fm1
....aauugcacuugucccgAccug.....................	1	1	Fm1
....aauugcacuugucccggccug.....................	389	0	Fm1
....aauugcacuugucAcggccugu....................	4	1	Fm1
....aauugcacuugucccggAcugu....................	112	1	Fm1
....aauugcacuugucccggccGgu....................	1	1	Fm1
....aauugcacuugucccggUcugu....................	76	1	Fm1
....aauugcUcuugucccggccugu....................	3	1	Fm1
....Uauugcacuugucccggccugu....................	13	1	Fm1
....aauugcacuugucccggcUugu....................	2	1	Fm1
....aauugcacuuAucccggccugu....................	20	1	Fm1
....aauugcacuugucccgAccugu....................	30	1	Fm1
....aauugcacuuguccAggccugu....................	1	1	Fm1
....aauugcacuugucccggccAgu....................	1	1	Fm1
....aauugcacuugucccgCccugu....................	30	1	Fm1
....aauugcacuugucccggccuUu....................	4	1	Fm1
....aauuAcacuugucccggccugu....................	2	1	Fm1
....aauugcacuugucccggccugC....................	38	1	Fm1
....aauugcacuuguUccggccugu....................	9	1	Fm1
....aauugcacuugucGcggccugu....................	4	1	Fm1
....aauugcacuugucUcggccugu....................	9	1	Fm1
....aauugcacuugucccggccugG....................	256	1	Fm1
....aCuugcacuugucccggccugu....................	1	1	Fm1
....aauugcacCugucccggccugu....................	2	1	Fm1
....aaAugcacuugucccggccugu....................	5	1	Fm1
....Cauugcacuugucccggccugu....................	2	1	Fm1
....aauugcacuugucccggccugA....................	18	1	Fm1
....aauugcaUuugucccggccugu....................	2	1	Fm1
....aauugcacuugucccggGcugu....................	47	1	Fm1
....aauugcGcuugucccggccugu....................	6	1	Fm1
....aauugcacuugucccgUccugu....................	25	1	Fm1
....aauugcacuugucccggccuCu....................	12	1	Fm1
....aauugcacuuguAccggccugu....................	2	1	Fm1
....aaNugcacuugucccggccugu....................	1	1	Fm1
....aaGugcacuugucccggccugu....................	1	1	Fm1
....aauugcacuugucccggccuAu....................	1	1	Fm1
....Gauugcacuugucccggccugu....................	2	1	Fm1
....aauCgcacuugucccggccugu....................	6	1	Fm1
....aauugcacuCgucccggccugu....................	4202	1	Fm1
....aauugcacAugucccggccugu....................	3	1	Fm1
....aauugcacuugucccggcAugu....................	3	1	Fm1
....aauugcacuugucccggccCgu....................	3	1	Fm1
....aGuugcacuugucccggccugu....................	3	1	Fm1
....aauugcacuuUucccggccugu....................	7	1	Fm1
....aauugcacuuguGccggccugu....................	1	1	Fm1
....aauuCcacuugucccggccugu....................	2	1	Fm1
....aauugAacuugucccggccugu....................	3	1	Fm1
....aauAgcacuugucccggccugu....................	5	1	Fm1
....aauugUacuugucccggccugu....................	4	1	Fm1
....aaCugcacuugucccggccugu....................	1	1	Fm1
....aauugNacuugucccggccugu....................	2	1	Fm1
....aauugcacuugucccggccugu....................	12699	0	Fm1
....aauugcacuuCucccggccugu....................	10	1	Fm1
....aauugcacuugCcccggccugu....................	1	1	Fm1
....aauugcacuugucccggcGugu....................	2	1	Fm1
....aauuUcacuugucccggccugu....................	1	1	Fm1
....Nauugcacuugucccggccugu....................	8	1	Fm1
....aauugcacuuguccUggccugu....................	6	1	Fm1
....aauugcacuugucccAgccugu....................	4	1	Fm1
....aauugcacuugucccggccuUuu...................	1	1	Fm1

```
                                  Star                                    Mature
uuggaguuggacuuucacuuuugucauguugggcaggcugugaugggugcaaaucuguaucaucaauuagaauugcacuugucccggccuguuugauugacugcaguucauc
......................................................................aauugcacuugucccggUcuguu...................   12     1   Fm1
......................................................................aauugcacuugucccgAccuguu...................   4      1   Fm1
......................................................................aauugcacuugucccggccuguC...................   1      1   Fm1
......................................................................aauugcacuuUucccggccuguu...................   3      1   Fm1
......................................................................aauugcacuugucccgUccuguu...................   6      1   Fm1
......................................................................aauugcacuugucccggAcuguu...................   26     1   Fm1
......................................................................aauugcacuugucccggccuCuu...................   1      1   Fm1
......................................................................aauugcacuugucccUgccuguu...................   1      1   Fm1
......................................................................aauugcacuugucccggccuguG...................   31     1   Fm1
......................................................................aauugcacuugucccggccuAuu...................   1      1   Fm1
......................................................................aauugcacuugucccgCccuguu...................   2      1   Fm1
......................................................................aauugNacuugucccggccuguu...................   1      1   Fm1
......................................................................aauuAcacuugucccggccuguu...................   1      1   Fm1
......................................................................aauugcacuugucccggccugCu...................   2      1   Fm1
......................................................................aUuugcacuugucccggccuguu...................   1      1   Fm1
......................................................................aauugcacuugucccggGcuguu...................   5      1   Fm1
......................................................................aauugcacuugucUcggccuguu...................   1      1   Fm1
......................................................................aauugcGcuugucccggccuguu...................   1      1   Fm1
......................................................................aauugcacuuCucccggccuguu...................   2      1   Fm1
......................................................................aauugcacuuAucccggccuguu...................   9      1   Fm1
......................................................................aauugUacuugucccggccuguu...................   2      1   Fm1
......................................................................Nauugcacuugucccggccuguu...................   1      1   Fm1
......................................................................aauugcacuCgucccggccuguu...................   1117   1   Fm1
......................................................................aauugcacuuguUccggccuguu...................   1      1   Fm1
......................................................................aauugcacuugucccggccuguu...................   1931   0   Fm1
......................................................................aauCgcacuugucccggccuguu...................   1      1   Fm1
......................................................................aauugcacuugucccggccuguA...................   1163   1   Fm1
......................................................................aauugcacuugucccggAcuguuu..................   6      1   Fm1
......................................................................aauugcacuCgucccggccuguuu..................   357    1   Fm1
......................................................................aauugcacuugucAcggccuguuu..................   1      1   Fm1
......................................................................aauugcacuugucccgUccuguuu..................   1      1   Fm1
......................................................................aaCugcacuugucccggccuguuu..................   1      1   Fm1
......................................................................aauugcacuugucccggccuguuG..................   2      1   Fm1
......................................................................aauugcacuugucccggccuguuu..................   434    0   Fm1
......................................................................aauugcacuugucccgCccuguuu..................   2      1   Fm1
......................................................................Nauugcacuugucccggccuguuu..................   1      1   Fm1
......................................................................aauugcacuugucccgAccuguuu..................   1      1   Fm1
......................................................................aauugcacuugucccggGcuguuu..................   2      1   Fm1
......................................................................aauugcacuugucccggUcuguuu..................   3      1   Fm1
......................................................................aauugcacuugucccggccuguuA..................   23     1   Fm1
......................................................................aauugcacuugucccggccugCuu..................   1      1   Fm1
......................................................................aauugcacuuUucccggccuguuu..................   1      1   Fm1
......................................................................aauugcacuugucccggccuguAu..................   283    1   Fm1
......................................................................aauugcacuuAucccggccuguuu..................   1      1   Fm1
......................................................................aauugcacuugucccggccuguuuU.................   29     1   Fm1
......................................................................aauugcacuugucccggccuguuuA.................   1      1   Fm1
.......................................................................auugcacuugucccggccugG....................   1      1   Fm1
.......................................................................auugcacuugucccggGcugu....................   1      1   Fm1
.......................................................................auugcacuugucccggccugu....................   28     0   Fm1
.......................................................................auugcacuCgucccggccugu....................   8      1   Fm1
.......................................................................auugcacuuUucccggccugu....................   1      1   Fm1
.......................................................................auugcacuuguccUggccuguu...................   1      1   Fm1
.......................................................................auugcacuCgucccggccuguu...................   3      1   Fm1
.......................................................................auugcacuugucccggccuguA...................   11     1   Fm1
.......................................................................auugcacuugucccggccuguu...................   4      0   Fm1
.......................................................................auugcacuCgucccggccuguuu..................   2      1   Fm1
.......................................................................auugcacuugucccggccuguAu..................   5      1   Fm1
........................................................................uugcacuCgucccggccugu....................   2      1   Fm1
..........................................................................gcacuugucccggccugu....................   4      0   Fm1

..................................aggcugugaugggugcaaaucu........................................................   5      0   Mm3
....................................................................aAaauugcacuugucccggccuguu...................   1      1   Mm3
.....................................................................Caauugcacuugucccggccugu....................   14     1   Mm3
.....................................................................Caauugcacuugucccggccuguu...................   2      1   Mm3
......................................................................aauugcacuugucccggc........................   8      0   Mm3
......................................................................Nauugcacuugucccggc........................   1      1   Mm3
......................................................................aauugcacuugucccggcU.......................   1      1   Mm3
......................................................................aauugcacuugucccggcc.......................   28     0   Mm3
......................................................................aauugcacuugucccggcA.......................   1      1   Mm3
......................................................................aauugcacuuguccUggcc.......................   1      1   Mm3
```

Star Mature

```
uuggaguuggacuuucacuuuugucauguugggcaggcugugaugggugcaaaucuguaucaucaauuagaauugcacuugucccggccuguuugauugacugcaguucauc
......................................................................aauugcacuugucccggccG......................   10     1   Mm3
......................................................................aauugcacuugucccggGcu......................   1      1   Mm3
......................................................................aauugcacuAgucccggccu......................   1      1   Mm3
......................................................................aauugcacuugucccggUcu......................   2      1   Mm3
......................................................................aauugcacuugucccggcUu......................   1      1   Mm3
......................................................................aauugcacuuAucccggccu......................   8      1   Mm3
......................................................................aauugcacuuCucccggccu......................   2      1   Mm3
......................................................................aauugcacuugucccgCccu......................   3      1   Mm3
......................................................................aauugcacuugucccggAcu......................   2      1   Mm3
......................................................................aauugcacuugucccggccC......................   3      1   Mm3
......................................................................aauugcacuugucccgUccu......................   1      1   Mm3
......................................................................aauugcacuugucccggccA......................   2      1   Mm3
......................................................................aauugcacuugucccggccu......................   379    0   Mm3
......................................................................aauugcacuuUucccggccug.....................   7      1   Mm3
......................................................................aauugcacuugucccggccuC.....................   1      1   Mm3
......................................................................aauugcacuugucccggUcug.....................   5      1   Mm3
......................................................................aauugcacuugucccggccGg.....................   13     1   Mm3
......................................................................aauugAacuugucccggccug.....................   1      1   Mm3
......................................................................aGuugcacuugucccggccug.....................   1      1   Mm3
......................................................................aauugcacuugucccggccCg.....................   11     1   Mm3
......................................................................aauugcacuugucccgCccug.....................   1      1   Mm3
......................................................................aauugcacuugucccggAcug.....................   11     1   Mm3
......................................................................aauugcacuugucccgUccug.....................   4      1   Mm3
......................................................................aauugcacuugucccggccug.....................   748    0   Mm3
......................................................................aauugcacuugucccggGcug.....................   7      1   Mm3
......................................................................aauugcacuugucccgAccug.....................   3      1   Mm3
......................................................................aauugcacuuAucccggccug.....................   1      1   Mm3
......................................................................aauugcacuugucccggccuA.....................   2      1   Mm3
......................................................................aauugcacuugucccggccuU.....................   5      1   Mm3
......................................................................aauugcacuuguUccggccug.....................   3      1   Mm3
......................................................................aauugNacuugucccggccugu....................   3      1   Mm3
......................................................................aauugAacuugucccggccugu....................   2      1   Mm3
......................................................................aauugcacuuguccUggccugu....................   24     1   Mm3
......................................................................aauugcacuuAucccggccugu....................   96     1   Mm3
......................................................................aauugcacuCgucccggccugu....................   6582   1   Mm3
......................................................................aauugcacuugucccggccugG....................   434    1   Mm3
......................................................................aaAugcacuugucccggccugu....................   10     1   Mm3
......................................................................aauugcacAugucccggccugu....................   5      1   Mm3
......................................................................aauugcacuugucccCgccugu....................   5      1   Mm3
......................................................................aaCugcacuugucccggccugu....................   8      1   Mm3
......................................................................aaGugcacuugucccggccugu....................   6      1   Mm3
......................................................................aauCgcacuugucccggccugu....................   5      1   Mm3
......................................................................Cauugcacuugucccggccugu....................   3      1   Mm3
......................................................................aauugcacuugucccgAccugu....................   110    1   Mm3
......................................................................aauugcacuAgucccggccugu....................   5      1   Mm3
......................................................................aauugcacuuUucccggccugu....................   115    1   Mm3
......................................................................aauugcacuugAcccggccugu....................   1      1   Mm3
......................................................................aCuugcacuugucccggccugu....................   6      1   Mm3
......................................................................aauAgcacuugucccggccugu....................   3      1   Mm3
......................................................................aauugcacuugucccggcAugu....................   2      1   Mm3
......................................................................aauugcacuugucccggccCgu....................   5      1   Mm3
......................................................................aauugcaGuugucccggccugu....................   1      1   Mm3
......................................................................aauuUcacuugucccggccugu....................   7      1   Mm3
......................................................................aauugcacuuguccAggccugu....................   3      1   Mm3
......................................................................aauugcacuugucccggAcugu....................   371    1   Mm3
......................................................................aauugcacuugucccggccugC....................   328    1   Mm3
......................................................................aauugcacuugucccggccAgu....................   1      1   Mm3
......................................................................aauugcGcuugucccggccugu....................   9      1   Mm3
......................................................................aauugcacuuguAccggccugu....................   5      1   Mm3
......................................................................aauugcacuugucccggccuUu....................   11     1   Mm3
......................................................................aauugcaUuugucccggccugu....................   8      1   Mm3
......................................................................aauugcacuugucccggUcugu....................   227    1   Mm3
......................................................................aauugcacuugucAcggccugu....................   8      1   Mm3
......................................................................aauugcacuugucGcggccugu....................   7      1   Mm3
......................................................................aGuugcacuugucccggccugu....................   14     1   Mm3
......................................................................aauugcNcuugucccggccugu....................   3      1   Mm3
......................................................................aauugcacuugCcccggccugu....................   3      1   Mm3
......................................................................aauugcacuugucccgUccugu....................   96     1   Mm3
......................................................................aauugUacuugucccggccugu....................   10     1   Mm3
......................................................................aauugcUcuugucccggccugu....................   8      1   Mm3
```

```
                                                 Star                                  Mature
uuggaguuggacuuucacuuuugucauguugggcaggcugugaugggugcaaaucuguaucaucaauuagaauugcacuugucccggccuguuugauugacugcaguucauc
......................................................................aauugcacuugucccAgccugu....................  10     1   Mm3
......................................................................aauugcacuugucccgCccugu....................  114    1   Mm3
......................................................................aauugcacuuguGccggccugu....................  2      1   Mm3
......................................................................aUuugcacuugucccggccugu....................  9      1   Mm3
......................................................................aauuCcacuugucccggccugu....................  1      1   Mm3
......................................................................aauugcacCugucccggccugu....................  1      1   Mm3
......................................................................aauugcacuugucccggccuAu....................  3      1   Mm3
......................................................................aauugcacuugucccggcUugu....................  6      1   Mm3
......................................................................aauuAcacuugucccggccugu....................  3      1   Mm3
......................................................................aauugcacuuguUccggccugu....................  21     1   Mm3
......................................................................aauugcacuugucccggGcugu....................  164    1   Mm3
......................................................................aauugcacuugucccggccugA....................  70     1   Mm3
......................................................................aauugcacuuguccGggccugu....................  3      1   Mm3
......................................................................aauugcacuGgucccggccugu....................  1      1   Mm3
......................................................................aauugcacGugucccggccugu....................  3      1   Mm3
......................................................................Nauugcacuugucccggccugu....................  15     1   Mm3
......................................................................aauugcacuugucccggccugu....................  43909  0   Mm3
......................................................................aauugcacuugucccggccGgu....................  3      1   Mm3
......................................................................aNuugcacuugucccggccugu....................  1      1   Mm3
......................................................................Gauugcacuugucccggccugu....................  6      1   Mm3
......................................................................aauugcaAuugucccggccugu....................  1      1   Mm3
......................................................................aaNugcacuugucccggccugu....................  1      1   Mm3
......................................................................Uauugcacuugucccggccugu....................  53     1   Mm3
......................................................................aauugcacuugucccUgccugu....................  4      1   Mm3
......................................................................aauugcacuuCucccggccugu....................  45     1   Mm3
......................................................................aauGgcacuugucccggccugu....................  1      1   Mm3
......................................................................aauugcacuugucccggccuCu....................  42     1   Mm3
......................................................................aauugcCcuugucccggccugu....................  2      1   Mm3
......................................................................aauugcacuugucUcggccugu....................  23     1   Mm3
......................................................................Gauugcacuugucccggccuguu...................  1      1   Mm3
......................................................................aauugcacuuguccUggccuguu...................  2      1   Mm3
......................................................................aauugcacuAgucccggccuguu...................  1      1   Mm3
......................................................................aauugcacuugucccggccuguC...................  2      1   Mm3
......................................................................aauugAacuugucccggccuguu...................  1      1   Mm3
......................................................................aauugcacuugucccggccuguu...................  4307   0   Mm3
......................................................................aauCgcacuugucccggccuguu...................  1      1   Mm3
......................................................................aauugcacuugucccgUccuguu...................  9      1   Mm3
......................................................................aauuUcacuugucccggccuguu...................  1      1   Mm3
......................................................................aauugcacuuUucccggccuguu...................  9      1   Mm3
......................................................................aauugcacuugAcccggccuguu...................  1      1   Mm3
......................................................................aauugcacuugucccggccuguG...................  20     1   Mm3
......................................................................aauugcacuugucccggcAuguu...................  1      1   Mm3
......................................................................aauugcacuGgucccggccuguu...................  1      1   Mm3
......................................................................aauugcacuugucUcggccuguu...................  3      1   Mm3
......................................................................aauugcacuugucccggccuguA...................  2392   1   Mm3
......................................................................aauugcacuugucccAgccuguu...................  2      1   Mm3
......................................................................aauugcaUuugucccggccuguu...................  1      1   Mm3
......................................................................aauugcGcuugucccggccuguu...................  5      1   Mm3
......................................................................aauugcacuuguccAggccuguu...................  1      1   Mm3
......................................................................aaAugcacuugucccggccuguu...................  1      1   Mm3
......................................................................aaCugcacuugucccggccuguu...................  1      1   Mm3
......................................................................aauugcacuugucAcggccuguu...................  1      1   Mm3
......................................................................aauugcaAuugucccggccuguu...................  1      1   Mm3
......................................................................aauugcacuugucccggccuCuu...................  5      1   Mm3
......................................................................aGuugcacuugucccggccuguu...................  3      1   Mm3
......................................................................aauugcacAugucccggccuguu...................  1      1   Mm3
......................................................................aauugcacuugucccggGcuguu...................  17     1   Mm3
......................................................................aUuugcacuugucccggccuguu...................  3      1   Mm3
......................................................................aauugUacuugucccggccuguu...................  1      1   Mm3
......................................................................aauugcacuuAucccggccuguu...................  8      1   Mm3
......................................................................aauugcacuugucccggccugGu...................  1      1   Mm3
......................................................................aauugcacuugucccgCccuguu...................  9      1   Mm3
......................................................................aauugcacuugucccggUcuguu...................  22     1   Mm3
......................................................................aauugcacuugucccggccugCu...................  33     1   Mm3
......................................................................aauugcacuugucccggAcuguu...................  41     1   Mm3
......................................................................aauugcacuuCucccggccuguu...................  4      1   Mm3
......................................................................aauugcaGuugucccggccuguu...................  1      1   Mm3
......................................................................Uauugcacuugucccggccuguu...................  8      1   Mm3
......................................................................aauugcacuugucccgAccuguu...................  17     1   Mm3
......................................................................aauugcacuugucccggccuUuu...................  2      1   Mm3
```

Star Mature

```
uuggaguuggacuuucacuuuugucauguugggcaggcugugaugggugcaaaucuguaucaucaauuagaauugcacuugucccggccuguuugauugacugcaguucauc
......................................................................aauugcacuCgucccggccuguu...................   861   1   Mm3
......................................................................aauugcacuugucccggccugAu...................   1     1   Mm3
......................................................................aauugcacuugucccggccuAuu...................   2     1   Mm3
......................................................................Nauugcacuugucccggccuguu...................   5     1   Mm3
......................................................................aauugcacuugucccgAccuguuu..................   4     1   Mm3
......................................................................aauugcacuugucccggccuguuA..................   36    1   Mm3
......................................................................aaGugcacuugucccggccuguuu..................   1     1   Mm3
......................................................................aauugcacuugucccggAcuguuu..................   7     1   Mm3
......................................................................aauugcacuugucccggccugAuu..................   1     1   Mm3
......................................................................aauugcacuugucccggccuguuu..................   846   0   Mm3
......................................................................aauugcacuuguGccggccuguuu..................   1     1   Mm3
......................................................................aauugcacuugucccggccuguuG..................   5     1   Mm3
......................................................................aauugcacuugucccggccuguuC..................   1     1   Mm3
......................................................................aUuugcacuugucccggccuguuu..................   1     1   Mm3
......................................................................aauugcacuugucccggGcuguuu..................   2     1   Mm3
......................................................................aauugcacuugucccggccugCuu..................   1     1   Mm3
......................................................................aauugcacuugucAcggccuguuu..................   1     1   Mm3
......................................................................aauugcacuuUucccggccuguuu..................   5     1   Mm3
......................................................................aauugcacuugucccggUcuguuu..................   3     1   Mm3
......................................................................aauugcacuugucccggccuguGu..................   2     1   Mm3
......................................................................aauugcacuugucccggccuguAu..................   931   1   Mm3
......................................................................aauugcacuuAucccggccuguuu..................   1     1   Mm3
......................................................................aaCugcacuugucccggccuguuu..................   1     1   Mm3
......................................................................aauugcacuugucccgCccuguuu..................   1     1   Mm3
......................................................................aauugcacuCgucccggccuguuu..................   220   1   Mm3
......................................................................aauugcacuugucccggccuguuuU.................   55    1   Mm3
.......................................................................auuAcacuugucccggccu......................   1     1   Mm3
.......................................................................auugcacuugucccggccCg.....................   1     1   Mm3
.......................................................................auugcacuugucccggccug.....................   1     0   Mm3
.......................................................................auugcacuugCcccggccugu....................   1     1   Mm3
.......................................................................auugcacuuUucccggccugu....................   3     1   Mm3
.......................................................................auugcaGuugucccggccugu....................   1     1   Mm3
.......................................................................auugcacuCgucccggccugu....................   19    1   Mm3
.......................................................................auugcacuugucccggccugu....................   102   0   Mm3
.......................................................................auugcacuugucccggccugC....................   1     1   Mm3
.......................................................................auugcacuugucccggccuUu....................   2     1   Mm3
.......................................................................auugcacCugucccggccugu....................   1     1   Mm3
.......................................................................auugcacuuUucccggccuguu...................   1     1   Mm3
.......................................................................auugcacuugucccggccuguu...................   14    0   Mm3
.......................................................................auugcacuugucccggccuguA...................   17    1   Mm3
.......................................................................auugcacuCgucccggccuguu...................   2     1   Mm3
.......................................................................auugcacuugucccggccuguAu..................   15    1   Mm3
.......................................................................auugcacuugucccggccuguuu..................   1     0   Mm3
........................................................................uugcacuugucccggccug.....................   2     0   Mm3
........................................................................uugcacuugucccggccugu....................   2     0   Mm3
........................................................................uugcacuugucccggccuguu...................   1     0   Mm3
.........................................................................ugcacuugucccggccug.....................   1     0   Mm3
.........................................................................ugcacuugucccggccuguu...................   1     0   Mm3
..........................................................................gcacuugucccggccugu....................   8     0   Mm3
..........................................................................gcacuugucccggccuguA...................   1     1   Mm3
..........................................................................gcacuugucccggccuguu...................   1     0   Mm3
............................................................................acuugucccggccuguuu..................   1     0   Mm3
............................................................................acuugucccggccuguAu..................   1     1   Mm3

..................................aggcugugauAggugcaaaucu........................................................   1     1   Fm3
..................................aggcugugaugggugcaaaucu........................................................   6     0   Fm3
.....................................................................Caauugcacuugucccggccugu....................   1     1   Fm3
......................................................................aauugcacuugucccggc........................   3     0   Fm3
......................................................................aauugcacuugucccggcc.......................   12    0   Fm3
......................................................................aauugcacuugucccggcA.......................   1     1   Fm3
......................................................................aauugcacuugucccggccu......................   35    0   Fm3
......................................................................aauugcacuuCucccggccu......................   1     1   Fm3
......................................................................aauugcacuuAucccggccu......................   1     1   Fm3
......................................................................aauugcacuugucccggUcug.....................   4     1   Fm3
......................................................................aauugcacuugucccggcAug.....................   1     1   Fm3
......................................................................aauugcacuuguUccggccug.....................   1     1   Fm3
......................................................................aauugcacuuAucccggccug.....................   3     1   Fm3
......................................................................aauugGacuugucccggccug.....................   1     1   Fm3
......................................................................aauugcacuuUucccggccug.....................   1     1   Fm3
......................................................................aauugcacuugucccggccuA.....................   1     1   Fm3
```

Star Mature

uuggaguuggacuuucacuuuugucauguugggcaggcugugaugggugcaaaucuguaucaucaauuagaauugcacuugucccggccuguuugauugacugcaguucauc			
....Gauugcacuugucccggccug.....................	2	1	Fm3
....aauugcacuGgucccggccug.....................	3	1	Fm3
....aauugcacuugucccgUccug.....................	1	1	Fm3
....aauugcacuugucccggGcug.....................	3	1	Fm3
....Uauugcacuugucccggccug.....................	2	1	Fm3
....aauugcacuugucccggccug.....................	882	0	Fm3
....aauugcacuugucccggAcug.....................	8	1	Fm3
....aauugcacuugucccgCccug.....................	2	1	Fm3
....aauugcacuugucccgAccug.....................	1	1	Fm3
....aaCugcacuugucccggccug.....................	1	1	Fm3
....aauugcacuugucccggccugG....................	5	1	Fm3
....Gauugcacuugucccggccugu....................	2	1	Fm3
....aauugGacuugucccggccugu....................	4	1	Fm3
....aaCugcacuugucccggccugu....................	5	1	Fm3
....Nauugcacuugucccggccugu....................	7	1	Fm3
....aauugcacCugucccggccugu....................	1	1	Fm3
....aauugcacuugucUcggccugu....................	12	1	Fm3
....aauugcacuugucGcggccugu....................	1	1	Fm3
....aauugcacuugucccUgccugu....................	2	1	Fm3
....aauugcacuAgucccggccugu....................	6	1	Fm3
....aauGgcacuugucccggccugu....................	1	1	Fm3
....aUuugcacuugucccggccugu....................	4	1	Fm3
....aauugcacuugAcccggccugu....................	2	1	Fm3
....aGuugcacuugucccggccugu....................	9	1	Fm3
....aauugcacuugucccgAccugu....................	45	1	Fm3
....aauugcacuuUucccggccugu....................	11	1	Fm3
....aauugcacuugucccggccugu....................	15412	0	Fm3
....aauugUacuugucccggccugu....................	3	1	Fm3
....aauuAcacuugucccggccugu....................	2	1	Fm3
....aauugcacuCgucccggccugu....................	6275	1	Fm3
....aauugcacuugucccggGcugu....................	46	1	Fm3
....aauugcacuugucAcggccugu....................	7	1	Fm3
....aauugcaUuugucccggccugu....................	1	1	Fm3
....aauugcacuuguGccggccugu....................	3	1	Fm3
....aauugcacuugucccggUcugu....................	71	1	Fm3
....aauugcacuugucccgUccugu....................	40	1	Fm3
....aauugcacuugCcccggccugu....................	2	1	Fm3
....aauuCcacuugucccggccugu....................	1	1	Fm3
....aauugcacuuCucccggccugu....................	15	1	Fm3
....aauugcacuugucccggccugC....................	40	1	Fm3
....aauugcacuuguAccggccugu....................	5	1	Fm3
....aauugcacuugucccgCccugu....................	27	1	Fm3
....aauCgcacuugucccggccugu....................	6	1	Fm3
....aauugcacuugucccggccuUu....................	1	1	Fm3
....aauugcacuugucccggccugA....................	58	1	Fm3
....aauugAacuugucccggccugu....................	2	1	Fm3
....aauugcGcuugucccggccugu....................	11	1	Fm3
....aauugcacuuguccUggccugu....................	22	1	Fm3
....aauugcUcuugucccggccugu....................	6	1	Fm3
....aauugcacuugucccggccuAu....................	4	1	Fm3
....aauugcacuugucccggcAugu....................	2	1	Fm3
....Cauugcacuugucccggccugu....................	3	1	Fm3
....aCuugcacuugucccggccugu....................	3	1	Fm3
....aauAgcacuugucccggccugu....................	4	1	Fm3
....aauugNacuugucccggccugu....................	7	1	Fm3
....aauugcacuugucccggccuCu....................	1	1	Fm3
....aauugcacuuguccGggccugu....................	2	1	Fm3
....aauugcacuugucccggccCgu....................	3	1	Fm3
....aauugcacuuguUccggccugu....................	10	1	Fm3
....aaGugcacuugucccggccugu....................	3	1	Fm3
....aauugcaAuugucccggccugu....................	1	1	Fm3
....aauugcacuuguccAggccugu....................	4	1	Fm3
....aauugcacuugucccggcGugu....................	1	1	Fm3
....aauugcCcuugucccggccugu....................	2	1	Fm3
....aauuUcacuugucccggccugu....................	5	1	Fm3
....aauugcacuugucccggcUugu....................	2	1	Fm3
....aauugcacuugucccggAcugu....................	133	1	Fm3
....Uauugcacuugucccggccugu....................	29	1	Fm3
....aauugcacuuAucccggccugu....................	31	1	Fm3
....aauugcacuugucccAgccugu....................	13	1	Fm3

Star **Mature**

uuggaguuggacuuucacuuuugucauguugggcaggcugugaugggugcaaaucuguaucaucaauuagaauugcacuugucccggccuguuugauugacugcaguucauc			
....aauugcacuugucGcggccuguu...................	1	1	Fm3
....aauugcacuugucccggGcuguu...................	3	1	Fm3
....aauugcacuuAucccggccuguu...................	3	1	Fm3
....aauugcacuugucccgUccuguu...................	3	1	Fm3
....aauugcacuuguUccggccuguu...................	1	1	Fm3
....aauugNacuugucccggccuguu...................	1	1	Fm3
....aauugcacuugucccggUcuguu...................	9	1	Fm3
....aauugcacuugucccggAcuguu...................	5	1	Fm3
....aauugcacuugucUcggccuguu...................	3	1	Fm3
....aauugcacuuguGccggccuguu...................	1	1	Fm3
....aauugcacuugucccggccuguA...................	912	1	Fm3
....aauugcacuugucccgCccuguu...................	1	1	Fm3
....aauugcacuugucccggccuguu...................	1036	0	Fm3
....aauuUcacuugucccggccuguu...................	1	1	Fm3
....aauugcacuuCucccggccuguu...................	1	1	Fm3
....aauugcacuugucccggccuguC...................	1	1	Fm3
....aauugcacuugucccgAccuguu...................	1	1	Fm3
....aauugcGcuugucccggccuguu...................	1	1	Fm3
....aauugcacuuguAccggccuguu...................	1	1	Fm3
....Uauugcacuugucccggccuguu...................	2	1	Fm3
....aauugcacuCgucccggccuguu...................	266	1	Fm3
....aGuugcacuugucccggccuguu...................	1	1	Fm3
....aauugcacuuguccAggccuguu...................	1	1	Fm3
....aauugcacuugucccggccugCu...................	3	1	Fm3
....aauugcacuugucccggccuguG...................	8	1	Fm3
....aauugcacuAgucccggccuguu...................	1	1	Fm3
....aauugcacuugucccggccuguuA..................	4	1	Fm3
....aauugcacuugucccggccuguuu..................	86	0	Fm3
....aauugcacuuguccUggccuguuu..................	3	1	Fm3
....aauugcacuugucccggccuguGu..................	1	1	Fm3
....aauugcacuugucccggccuguAu..................	436	1	Fm3
....aauugcacuugucccggAcuguuu..................	2	1	Fm3
....aauugcacuugucccggccuguuC..................	2	1	Fm3
....aauugcacuCgucccggccuguuu..................	45	1	Fm3
....aauugcacuugucccggccuguuuU.................	3	1	Fm3
....auugcacuugucccggccug.....................	4	0	Fm3
....auugcacuugucccggccCgu....................	1	1	Fm3
....auugcacuugucccggcGugu....................	1	1	Fm3
....auugcacuuUucccggccugu....................	4	1	Fm3
....auugcacAugucccggccugu....................	1	1	Fm3
....auugcacuCgucccggccugu....................	51	1	Fm3
....auugcacuugucccggccugu....................	40	0	Fm3
....auugcacuugucccggccugC....................	1	1	Fm3
....auugcacuCgucccggccuguu...................	2	1	Fm3
....auugcacuugucccggccuguA...................	10	1	Fm3
....auugcacuugucccggccuguu...................	8	0	Fm3
....auugcacuugucccggAcuguu...................	1	1	Fm3
....auugcacuCgucccggccuguuu..................	1	1	Fm3
....auugcacuugucccggccuguuA..................	1	1	Fm3
....auugcacuugucccggccuguAu..................	4	1	Fm3
....uugcacuugucccggccugC....................	3	1	Fm3
....uugcacuugucccggccugu....................	1	0	Fm3
....uugcacuugucccggccuguu...................	2	0	Fm3
....ugcacuugucccggccugu....................	1	0	Fm3
